# Supplementary material for: Effectiveness and Safety of Ayurvedic Medicines in Type 2 Diabetes Mellitus Management: A Systematic Review and Meta-Analysis
Source: Front Pharmacol. 2022 Jun 8;13:821810. doi: 10.3389/fphar.2022.821810 (PMC9213670; doi:10.3389/fphar.2022.821810)
Supplement: Supplementary file 1 [file DataSheet1.zip › Supplementary Material/Forest and Funnel Plots/Zingiber officinale/FBG.pdf]

Zingiber officinale - FBG

Study	Intervention N	Intervention Mean	Intervention SD	Comparator N	Comparator Mean	Comparator SD	Mean Diff. with 95% CI	Weight (%)
Mahluji 2013	28	147.00	23.00	30	159.00	42.00	-12.00 [-29.61, 5.61]	27.43
Arablou 2014a, Arablou 2014b	33	121.90	37.40	30	145.00	68.40	-23.10 [-49.99, 3.79]	11.76
Azimi 2014, Azimi 2016	41	366.34	51.99	39	353.23	74.69	13.11 [-14.97, 41.19]	10.78
Mozaffari-Khosravi 2014, Talaei 2017, Talaei 2018	40	153.12	48.34	41	153.73	50.57	-0.61 [-22.16, 20.94]	18.30
Shidfar 2015	22	142.09	47.90	23	157.10	81.83	-15.01 [-54.42, 24.40]	5.47
Arzati 2017, Zarezadeh 2018	23	144.00	65.30	22	173.00	63.90	-29.00 [-66.77, 8.77]	5.96
Carvalho 2020	47	174.05	64.10	56	175.98	72.57	-1.93 [-28.62, 24.76]	11.93
Gholinezhad 2020	21	166.19	56.83	21	172.28	48.28	-6.09 [-37.98, 25.80]	8.36
**Overall**							-8.00 [-17.22, 1.23]	

Heterogeneity: $\tau^2 = 0.00$, $I^2 = 0.00\%$, $H^2 = 1.00$

Test of $\theta_i = \theta_j$: $Q(7) = 5.55$, $p = 0.59$

Test of $\theta = 0$: $z = -1.70$, $p = 0.09$

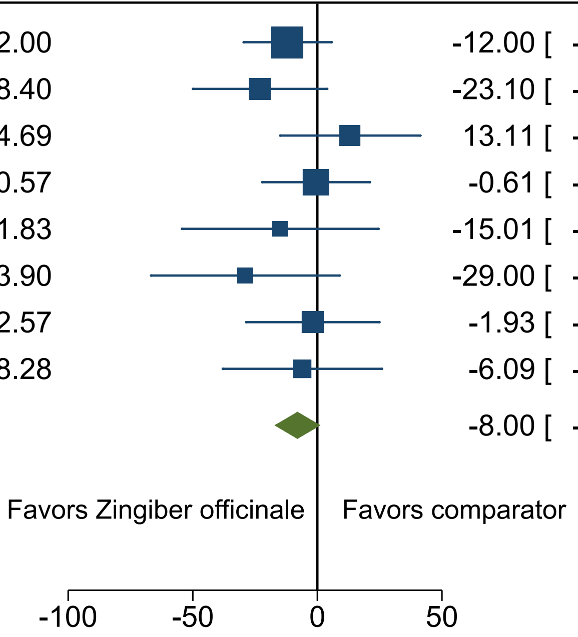

Random-effects REML model
